# Medical healthcare use in Parkinson's disease: survey in a cohort of ambulatory patients in Italy

**DOI:** 10.1186/1472-6963-5-26

**Published:** 2005-03-24

**Authors:** Marco Cosentino, Emilia Martignoni, Donatella Michielotto, Daniela Calandrella, Giulio Riboldazzi, Claudio Pacchetti, Gianmario Frigo, Giuseppe Nappi, Sergio Lecchini

**Affiliations:** 1 Department of Clinical Medicine, Section of Experimental and Clinical Pharmacology, University of Insubria, Varese, Italy; 2 Department of Medical Sciences, University of Piemonte Orientale "A. Avogadro", Novara and Neurorehabilitation and Movement Disorders Unit IRCCS "S. Maugeri" Scientific Institute of Veruno (NO), Italy; 3 Parkinson's Disease and Movement Disorders' Centre of Ospedale Di Circolo, Varese, Italy; 4 Parkinson's Disease and Movement Disorders' Centre of IRCCS C, Mondino, Pravia, Italy; 5 Department of Internal Medicine and Therapeutics, Section of Pharmacology, University of Pavia, Pavia, Italy; 6 Department of Neurology and Otorhinolaryngology, University "La Sapienza", Rome, Italy

## Abstract

**Background:**

Parkinson's disease (PD) is a chronic neurodegenerative disease which at present has no cure, and it usually results in severe disability. The burden of PD increases as the illness progresses, resulting in the extensive utilisation of both health and community services. Knowledge of healthcare use patterns and of their determinants may greatly contribute to improve patient care, however few studies have examined this issue in PD. The present study was devised to describe the type of and reasons for medical healthcare resource use in persons with PD attending a Centre for PD and Movement Disorders, and to examine drug prescriptions issued on such occasions.

**Methods:**

The study was a retrospective, cross-sectional survey in a cohort of ambulatory patients with PD, conducted by means of standard interviews.

**Results:**

In the year before the study, 92 (70.8%) of 130 patients used medical healthcare resources: 1/5 of the patients was admitted to hospital, 1/5 to emergency room, 2/5 were visited by a non-neurology specialist, and 1/4 by the GP. Reasons were: nearly 20% programmed hospital admissions and visits, and more than 25% injuries and musculo-skeletal diseases. Other conditions typically occurring in PD (e.g. dementia, diabetes and cardio- and cerebro-vascular disease) were less frequently involved. On such occasions, drugs for PD were occasionally changed, however drug prescriptions for other indications were issued to more than 66% of the patients.

**Conclusion:**

Several physicians other than the neurologist may take care of PD patients on different occasions, thus emphasising the need for communication between the reference neurologist and other physicians who from time to time may visit the patient.

## Background

Parkinson's disease (PD) is a neurodegenerative disease which at present has no cure, and, despite the variety of pharmacological and surgical treatment options [1-3], it usually results in severe disability. In Europe, age-adjusted prevalence rates of PD have been estimated at 1.6 per 100 population, with a steady increase in older groups, up to 3.5–3.6 in people aged 80 years and older [4]. Similar estimates have been reported for the United States [5]. In view of the increasing number of elderly people in developed countries, the prevalence of PD is expected to increase, as well.

Because PD is a chronic condition, the disease burden increases as the illness progresses, due to the appearance of both disease- and drug-related problems, resulting in the extensive utilisation of both health and community services [6-10]. The high rate of prevalent comorbid conditions occurring in PD, either associated or unrelated, significantly contributes to the utilisation of healthcare resources [11], which is higher than in subjects without PD [8] and has substantial economic implications [12]. As a consequence, it can be easily predicted that over the next few years more PD patients will use more healthcare resources, with a significant impact on the healthcare systems.

Knowledge of healthcare use patterns and of their determinants may greatly contribute to improve patient care, providing physicians, caregivers and politicians with information useful to estimate patient needs and to plan intervention strategies and allocation of resources. At present only few studies have examined healthcare use in PD patients and its relationship with potentially relevant factors, either related to the patient, such as age, sex and other social and demographic characteristics, or to the disease itself, such as severity, duration and comorbidity [8,11,13].

In the context of a project aimed at investigating healthcare needs, comorbidity, and drug use in PD patients, we previously reported that in PD patients attending a neurological service drug prescribing patterns may be associated with both disease- and patient-related factors, and that analysis of drug prescriptions may help to identify major comorbid conditions [14]. Since it was suggested that PD patients seen primarily by a neurologist may have an increased use of resources [15], as a logical extension of our previous work, we decided to investigate the use of healthcare resources in our cohort of ambulatory PD patients. However, as we were not interested in performing a formal cost-of-illness study, but rather to focus on the practical care of the patient, we directed our attention to medical healthcare resource use, with particular regard to the consequences for drug treatments. Regarding resource data collected, we decided to focus on hospital and emergency room (ER) admissions, and general practitioner (GP) and non-neurologist specialist visits, as these are the circumstances when physicians other than the neurologist take care of PD patients.

Our specific aims were: to assess the reasons for medical healthcare use, to look for possible relationship between the medical healthcare use and the pattern of anti-Parkinson drug (APD) medications, and to assess drug prescriptions issued by other physicians. All these aspects have so far received little attention, despite their straightforward relevance for the care of PD patients.

## Methods

The present study is part of a cross-sectional survey of PD outpatients consecutively attending the Centre for PD and Movement Disorders of the Neurological Service of the Ospedale di Circolo of Varese (Italy). The diagnosis of PD was established by neurologists skilled in movement disorders, according to the United Kingdom Parkinson's Disease Society Brain Bank Criteria [16]. Parkinsonisms resulting from other degenerative conditions or secondary to drugs, metabolic disorders or exposure to toxins were excluded. All the participants in the study gave informed consent.

Information about medical healthcare use over the previous year were obtained during a standard interview with the patient and/or her/his caregiver(s), by using self-report questionnaires. Medical healthcare resources addressed included: hospital admissions, ER admissions, GP and/or non-neurologist specialist visits (excluding therefore programmed neurological visits directly related to the follow-up of PD). Information was specifically sought about: the number of times each medical healthcare resource was used, the reasons for use, the occurrence on such occasions of modifications of the APD treatment and the prescription of drugs for indications other than PD (non-APDs). Collected information also included: the main patient demographic and clinical data (i.e. sex, age, age at onset and duration of PD), severity of PD, scored according to the Hoehn and Yahr (H&Y) scale [17], information about current drug treatments.

Data were stored in a database for subsequent analysis. To ensure confidentiality, patients' names were not recorded and a unique personal identification code was used to perform data linkage. Reasons for medical healthcare resource use as well as indications for drug use were coded using the International Classification of Diseases, 9^th ^revision, Clinical Modifications (ICD-9-CM) [18], whereas drugs were classified according to the Anatomical, Therapeutic and Chemical (ATC) classification index [19].

### Statistics

Results are presented as mean ± SD (unless otherwise stated) with n indicating the number of observations. Analysis of the data was performed on the whole patient population and on groups of patients stratified according to: decades of age and age at onset of PD, years of PD duration, and H&Y stage. Statistical significance of the differences between groups was assessed by use of parametric (Student's t test or ANOVA followed by Tukey-Kramer Multiple Comparisons post test) or non-parametric (Kruskal-Wallis analysis followed by Dunn's Multiple Comparison test) tests, according to the results of a preliminary normality test. Frequency distribution differences were analysed using contingency tables and the χ^2 ^test or the Fisher's exact test, as appropriate. All the statistical calculations were performed by use of a commercially available statistical software (GraphPad Prism version 3.0 for Windows, GraphPad Software, San Diego California USA, ).

## Results

### Use of medical healthcare resources

The study included 130 persons with PD, 69 females and 61 males. Their characteristics are shown in Table 1. Fifty four patients (41.5% of total patients) had dyskinesia and 88 (67.7%) referred fluctuations of motor performances, including wearing off and on off phenomena, while 42 subjects (32.3%) were non fluctuators. Ten patients (7.7%) were taking antipsychotics for hallucinations, 22 (16.9%) were treated with antidepressants, 38 (29.2%) received anxiolytics. All these drugs were prescribed and managed by the neurologist of the Centre for PD. There was no significant difference between females and males with respect to any of the characteristics considered (data not shown). In the previous year, 92 out of 130 patients, corresponding to the 70.8% of all the patients, used one or more medical healthcare resources (1.9 ± 1.1 resources/patient, range 1–5). There was no significant difference between patients using and not using medical healthcare resources (Table 1). However, among patients using resources both age at onset of PD and PD duration were significantly different according to the type of resource used (Table 2). This finding was further supported by stratified analysis of the patients, inasmuch as 35.8% of the patients aged at onset of PD 60 years or more had at least a GP visit vs 12.2% in patients with earlier PD onset (*P *= 0.0040), and 41% of the patients with disease duration longer than 8 years were admitted to ER vs 13.5% of those with shorter disease duration (*P *= 0.0190). Other significant differences were: 75% of the patients aged more than 60 years used any type of resources vs. 50% of the younger ones (*P *= 0.0129), 35.4% of the patients aged 70 years or more and 50% of those in H&Y stage IV had at least a GP visit vs. 18.5% of younger patients (*P *= 0.0471) and 20.6% in those in lower stages (*P *= 0.0191).

**Table 1:** Characteristics of the patients

	Overall	Resource use	
		yes	no
patients, n (%)	130 (100)	92 (70.8)	38 (29.2)
age (years)	68.6 ± 10.0 (38–87)	69.4 ± 9.1 (45–86)	66.6 ± 11.7 (38–87)
age at onset of PD (years)	61.7 ± 10.2 (36–84)	62.3 ± 9.0 (40–84)	60.1 ± 12.7 (36–82)
PD duration (years)	6.9 ± 4.5 (1–24)	7.1 ± 4.8 (1–24)	6.5 ± 3.8 (1–14)
H&Y stage	2.6 ± 1.0 (I-IV)	2.7 ± 1.0 (I-IV)	2.5 ± 0.9 (I-IV)

Data are means ± SD (min-max), unless otherwise indicated.

**Table 2:** Characteristics of the patients according to the use of medical healthcare resources

	Hospital admissions	ER admissions	Specialist visits	GP visits	*P*
resources used, n	29	31	74	42	
patients, n (%)	25 (19.2)	29 (22.3)	54 (41.5)	35 (26.9)	
resources/patient, mean (range)	1.2 (1–3)	1.1 (1–2)	1.4 (1–3)	1.2 (1–3)	
patient characteristics					
age (years)	71.0 ± 9.4 (45–86)	68.1 ± 9.0 (47–83)	68.6 ± 9.1 (45–85)	72.9 ± 7.7 (54–85)	Ns
age at onset of PD (years)	64.3 ± 9.4 (40–84)	58.7 ± 7.3* (42–72)	62.0 ± 8.6 (40–80)	64.1 ± 7.5 (50–78)	0.037
PD duration (years)	6.7 ± 3.5 (2–20)	9.4 ± 3.8^# ^(2–20)	6.6 ± 3.7 (1–24)	8.8 ± 4.6 (1–24)	0.004
H&Y stage	2.8 ± 0.9 (I-IV)	3.0 ± 1.0 (I-IV)	2.5 ± 1.0 (I-IV)	3.0 ± 1.0 (I-IV)	Ns

Data are means ± SD (min-max), unless otherwise indicated.

* = *P *< 0.05 vs GP visits; # = *P *< 0.05 vs specialist visits.

As regards patient gender, there were no major differences. However, stratified analysis revealed minor differences, as described hereafter:

- the frequency of hospital admissions was significantly higher in males than in females in H&Y stage III (28.6% vs 3.8%, *P *= 0.0347) and in the age interval 70–79 years (33.3% vs 6.9%, *P *= 0.0407);

- among females hospital admissions were more common in patients in H&Y stage II (30.8% vs 7% of patients in other stages, *P *= 0.0405), while among males they were more frequent in H&Y stage IV (47.1% vs 11.4% in lower stages, *P *= 0.0194);

- in males (but not in females) the frequency of ER admissions and GP visits increased with increasing PD duration (52.6% and 42.1% of the male patients with disease duration longer than 8 years vs 9.5% and 11.9% with shorter disease duration, *P *= 0.0068 and *P *= 0.0048 respectively).

### Reasons for medical healthcare resource use

According to the ICD-9-CM classification [18], the most common causes for resource use were represented by **programmed hospital admissions and control visits**, which were included in the category 'supplementary classification of factors influencing health status and contact with health services'. The next most frequent category were **symptoms, signs and ill-defined conditions**, collecting several complaints which could not be precisely assigned to other more specific categories. **Injuries **were 23 cases in total and included 10 fractures and 11 ER admissions for injuries due to falls. Table 3 collects detailed information about all the reasons for medical healthcare resource use recorded in the present study.

**Table 3:** ICD-9-CM classification of the reasons for medical healthcare resource use

	Hospital admissions	ER admissions	Specialist visits	GP visits	Total	%
**factors influencing health status and contact with health services (V01–V83)**	rehabilitation (4)	=	rehabilitation (5); problems of eye (7), heart (3), diabetes (2), urogenital (2), skin (1); Rx of hip (1)	general control (8); urinary cathether substitution (1)	34	19.3
**symptoms, signs, and ill-defined conditions (780–799)**	chest pain (2); sleep disturbance (1); epistaxis (1); abdominal pain (1)	abdominal pain (2); dyskinesia (1); epistaxis (1); dysphagia (1)	abdominal pain (6); hallucination (1); headache (1)	abdominal pain (2); hallucination (1); cough (1); nausea, vomiting (1); urinary incontinence (1)	24	13.7
**injury (800–959)**	fracture of femur (1), femur plus humerus (1)	injuries due to falls (11); fractures: ribs (2), wrist (1), upper limb (1), foot (1), unspecified (2); open wound (1)	=	fracture unspecified (1); open wound (1)	23	13.1
**diseases of musculo-skeletal system and connective tissue (710–739)**	osteoarthrosis (1)	pain in joint (1); lumbago (1)	intervertebral discopathy (3); lumbago (3); pain in joint (2); osteoarthrosis (2); spondilosis (1); osteomyelitis (1)	pain in joint (4); lumbago (2); rheumatism (1)	22	12.5
**diseases of the circulatory system (390–459)**	acute hypotension (2); cerebral ischemia (1); aortic aneurysm (1)	=	hypertension (3); ischemic heart disease (2); atrial fibrillation (1); myocardial degeneration (1); aortic aneurysm (1); varicous veins (1)	hypotension (2); hypertenson (1)	16	9.1
**diseases of the nervous system and sense organs (320–389)**	cataract (3)	hearing loss (1)	tinnitus (1); presbyopia (1); cataract (1); hearing loss (2); glaucoma (1); impacted cerumen (1)	conjunctivitis (2); vertiginous syndrome (1)	14	7.9
**diseases of the digestive system (520–527)**	abdominal hernia (2); gastric ulcer (1); constipation (1); cholelithiasis (1)	pulp disease (1); teeth extraction (1)	avulsions of teeth (2); periodontal disease (1)	constipation (2); intestinal obstruction (1); cholelithiasis (1)	14	7.9
**diseases of respiratory system (460–519)**	pneumonia (1)	pneumonia (1)	asthma (1)	influenza (5); acute laringytis (1)	9	5.1
other*	4	1	13	2	20	11.4

**Total**	29	31	74	42	176	100.0

* = encompasses the following ICD-9-CM categories (which individually accounted for less than 5% of total medical healthcare resource use): neoplasms (140–239), n = 8, 4.5%; diseases of genitourinary system (580–629), 7, 4%; diseases of skin (680–709), 3, 1.7%; infectious and parasitic diseases (001–139), 1, 0.7%; mental diseases (290–319), 1, 0.7%.

### Relationship between use of medical healthcare resources and APD treatment patterns

Patients treated with levodopa alone or associated with other APDs were respectively 62 and 66, and they did not differ in the overall frequency of healthcare resource use (75.8% vs 65.1%). As regards the frequency of use of specific medical healthcare resources, more patients on levodopa alone had GP visits than those on levodopa associated with other APDs (37.1% vs 18.2%, *P *= 0.018). No significant differences were found in the frequency of hospital admissions (21.0% vs 16.7%), ER admissions (43.5% vs 39.4%), or specialist visits (21.0% vs 22.7%). The only two patients on DA agonists alone both used medical healthcare resources: one was admitted to hospital and to ER (on separate occasions), and the other had two different (and separate) specialist visits.

### Drug prescription during medical healthcare resource use

**APD treatments: **A total of 11 patients (8.5% of total patients) had modifications of their APD treatment, most often during hospital admissions (6 patients), followed by specialist visits (2), GP visits (1 patient) and ER admissions (1 patient). More than one third of hospital admissions (11 out of 29, 37.9%) therefore resulted in changes of APD treatment (6 dose increased, 2 dose decreased, 2 drug withdrawn, 1 new prescription), which on the contrary occurred only occasionally as a consequence of specialist visits (1 dose decreased, 2 new prescriptions) or GP visits (2 drug withdrawn). Levodopa was the drug most frequently changed (10 prescription changes, including 3 dose increased, 2 dose decreased, 2 new prescriptions, and 2 drug withdrawn), while on the whole prescriptions of DA agonists were changed on 7 occasions (3 dose increased, 1 dose decreased, 1 new prescription, and 2 drug withdrawn). On one occasion there were no information concenring the specific type of change. **Non-APD treatments: **Prescriptions of non-APDs were issued to 86 patients (66.1% of total patients), most often during ER admissions and GP visits (37 and 36 patients, respectively), followed by specialist visits (13 patients). Most prescribed drugs were (ATC first level): general antiinfectives (J, 15 patients), musculo-skeletal system drugs (M, 14 patients), drugs for the alimentary tract and metabolism and for the cardiovascular system (A and C, both 11 patients). Information about occasions in which non-APDs were prescribed are given in Table 4. Data about prescriptions of non APDs during hospital admissions were not analysed since nearly all the patients and/or their caregivers were unable to report affordable information concerning this particular issue.

**Table 4:** Non-APD prescriptions issued on the occasion of medical healthcare resource use

ATC (first level)	ER admissions	Specialist visits	GP visits
A – alimentary tract and metabolism	2 (2)	3 (2)	9 (7)
B – blood and blood forming organs	5 (5)	2 (2)	=
C – cardiovascular system	6 (6)	=	5 (5)
D – dermatologicals	2 (2)	=	1 (1)
G – genitourinary system and sex hormones	4 (3)	=	1 (1)
H – systemic hormonal preparations, excluding sex hormones	2 (2)	=	=
J – general antiinfectives for systemic use	6 (5)	4 (4)	7 (6)
L – antineoplastic and immunomodulating agents	=	3 (3)	=
M – musculo-skeletal system	6 (5)	5 (4)	7 (5)
N – nervous system	=	1 (1)	5 (5)
P – antiparasitic products, insecticides and repellents	=	=	=
R – respiratory system	2 (1)	=	5 (4)
S – sensory organs	2 (2)	=	2 (1)
V – various	1 (1)	1 (1)	=
Other	4 (4)	=	1 (1)

Total	41 (37)	15 (13)	43 (36)

Data expressed as number of prescriptions and as number of patients (in parentheses).

## Discussion

The results of this study allowed to describe the type of and reasons for medical healthcare use in patients with PD referring to a neurological service dedicated to movement disorders, and to examine the role of patient- and disease-related factors, with particular regard to the pattern of APD treatment. In addition, the changes of APD medications and non-APD prescriptions as consequences of medical healthcare use were also specifically analysed.

A few points need to be preliminarily discussed about our data. First, this was a survey of PD outpatients attending a service devoted to movement disorders. But it also means that only persons who were ambulant or able to reach an outpatient service have been considered, and subjects with H&Y score over IV, which means in the very advanced stages of the disease, have not been included. Second, data collection was accomplished by retrospective self-report of events occurring in the previous 12 months, and it is possible that some respondents had difficulty in accurately reporting such information, even if most of these patients are known to carefully keep the documents concerning their health. Being aware of this potential bias, we sought only information that could be expected to be recalled with some degree of accuracy, therefore excluding e.g. drug prescriptions in hospital. Moreover, whenever possible, we cross-compared reported information with patient records in our service (e.g., in the case of medical healthcare resource use for symptoms, signs and ill-defined conditions and for injuries) and concluded that only negligible, if any, bias was introduced by inaccuracy of collected information.

According to our results, the most utilised medical healthcare resource were specialist visits (41.5% of the patients), followed by GP visits (26.9%), ER admissions (22.3%), and hospital admissions (19.2%). Consultations and hospital admissions for reasons related to PD were *a priori *excluded from our survey. However, according to the records of our service during the previous year, only 14 patients (11.5% of total patients) were admitted to the hospital for reasons related to the disease and requiring more careful clinical observation and drug changes, while the mean year frequency of visits for PD was 2.1 (range: 1–7). Other studies reported on average 5.4 medical visits and 2.7 inpatient hospital days in 6 months [15], or 6.0 physician consultations (including neurologist and other physicians) per year, and no hospital days and ER visits [8]. De Boer et al. [13] showed that nearly half of the patients had at least a GP visit in the previous 6 months. In comparison our patients require less consultations by GPs or non-neurological specialists, possibly due to the preferential consultation of the Centre for PD and Movement Disorders of their reference. On the other side, they seem to use more frequently hospital and ER services, although without any apparent relationship with patient and/or disease characteristics. This finding was quite unexpected, since disease severity is usually pointed to as one of the major predictors of increased healthcare use in PD [13,20-22]. Another study [10] however reported that disease severity better predicted non medical rather than medical needs.

In the present study, differences according to patient and disease characteristics regarded mainly the specific type of resource used (Table 2). In particular, patients admitted to hospital or consulting GP were slightly older at onset of PD, while those admitted to ER had longer disease duration, suggesting that need for hospital and/or GP care is related mainly to age, while disease duration is rather involved in acute comorbid events leading to ER admission. Further support to this suggestion comes from the observation that the most frequent causes of ER admissions were injuries (19, 61.3% of total ER admissions), mainly fractures (7, 36.8% of total injuries), which are typical complications in the advanced stages of the disease [23]. A recent study [24] comparing PD with old-age versus middle-age onset evidenced greater motor impairment in subjects with old-age onset, pointing to the possible role of age and comorbidities. On the other hand another study [25] describes similar severity and disability in patients with onset of the disease before 50 years and in persons with PD onset after 50 years, giving relevance to social and psychosocial factors in contributing to the impairment of quality of life. A final consideration can concern the comparison of our data with the ones concerning healthcare utilisation by a sample of 5000 UK population aged 65–90, showing that almost 80% of them visited a GP at least once a year and overall, women used more ambulatory care services and men hospitalised more often [26]. Similar data concerning hospitalisation were found in our cases, although we were not able to confirm the tendency of female patients to seek more often GP assistance. Differences in the overall organisation of the National Healthcare System between Italy and UK might however account for such a discrepancy. Indeed, only in males we observed a correlation between PD duration and the frequency of ER admissions and GP visits, possibly in line with the gender differences in the clinical aspects of the disease, with more severe motor impairment and behavioural problems in men [27].

Although it is well established that PD is often complicated by a burden of comorbid conditions, ranging from psychiatric disturbances [28] to bladder dysfunctions [29], relatively few studies exist which address the issue of comorbidity in PD from a comprehensive point of view. In the present study, we selectively targeted only those comorbid conditions which resulted in the use of medical healthcare resources, to address specifically how comorbidity impacts on medical healthcare use in PD patients. As a consequence, our results reflect the pattern of comorbid diseases which are serious enough to warrant medical intervention, and therefore may have a major impact on the general patient care. In this regard, it is of interest that three categories of disease conditions, namely symptoms and signs and ill-defined conditions, injuries, and diseases of the musculo-skeletal system, account for nearly 40% of all reasons for resource use (Table 3). Symptoms, signs and ill-defined conditions include mainly conditions such as abdominal pain due to constipation, sleep disturbance, hallucination, headache, dyskinesia, dysphagia, all representing likely complications of the course of PD, as also falls and fractures and lumbago and pain in joint [1,3]. It follows that in these persons more than 1 out of 3 reasons for medical healthcare resource use is justified by PD itself together with its burden of complications, while other conditions typically occurring with high frequency in PD patients, such as dementia, diabetes and cardio- and cerebrovascular disease [11]. On the other hand, psychiatric disturbances (hallucinations) in this sample accounted for only 1 specialist and 1 GP consultation, which seem quite low figures, in comparison to the known frequency and severity of such ailment in PD patients [12,13]. We have no exhaustive explanations for this finding, however it cannot be excluded that the management of the patients by a PD-dedicated centre may contribute to minimize the problems related to the disease and its therapy.

In the present study, we found no apparent correlation between resource use and APD treatments, with the only exception of a positive correlation between levodopa medication and GP visits. However, we believe that this is a spurious association, due to the fact that in our cohort PD patients on levodopa in association with other APD drugs or on DA agonists alone were significantly younger and had an earlier onset of PD than patients on levodopa alone [14]. This finding is in line with the study by Diederich et al. [24], which was performed in a similar population of patients referring to a centre for movement disorders. The preferential use of association treatments, or even of therapy with DA agonists alone [30], is thought to prevent the motor complications associated with long-term levodopa treatment [31]. Interestingly however, in the present investigation no significant difference was found in medical healthcare use according to the various patterns of APD medication, i.e. levodopa alone, levodopa associated with other APD (mainly DA agonists), or DA agonists alone. It cannot be excluded that the fact that the present patient sample refers to a centre for PD and movement disorders may account for an optimisation of individual therapeutic schemes, possibly leading to a reduced risk of complications.

An additional objective of our investigation was the study of drug prescriptions on the occasions of medical healthcare resource use. According to our results, APD treatments were modified in 8.5% of the patients, mainly (55% of the cases) during hospital admissions. Levodopa was the drug most frequently modified (59% of the cases). Considering that in the present population APD treatments account for 307 prescriptions [14], the total number of prescriptions changed (17, 5.5% of total prescriptions) may be considered low. It seems thus that APD treatment remains primarily the responsibility of the neurologist. On the contrary, as for non-APD treatments we found that a total of 99 prescriptions were issued to 86 patients (66% of total patients), which look as very high figures. GP (43% of total prescriptions) and ER (41%) were the main responsible for such prescriptions. While prescriptions on the occasions of ER admissions are more likely to reflect acute and intercurrent needs, prescriptions by GP may reflect the need to treat chronic illness, although this speculation should be specifically addressed in ad hoc studies. Taking in mind that in our previous study [14] we found in this cohort of patients 252 non-APD prescriptions, the present results suggest that physicians other than the reference neurologist are responsible for a significant fraction of such prescriptions, and further emphasise the need for an accurate update of the patient files on the occasions of control visits, as well as for a continuous communication between the reference neurologist and other physicians who from time to time may take care of the patient.

## Conclusion

In summary, we have described the type of and reasons for medical healthcare use during 12 months in a cohort of patients with PD referring to a neurological service dedicated to movement disorders. According to our results, major determinants of resource use are programmed hospital admissions and visits, injuries and diseases of the musculo-skeletal system; new drug prescriptions (mainly non-APD drugs) are a common occurrence on the occasions of resource use. Optimisation of the global assistance to patients, including prevention of traumatic accidents, could therefore result effective to reduce and rationalise resource use. The need for careful periodical check of drug treatments is also emphasised.

## Competing interests

The author(s) declare that they have no competing interests.

## Authors' contributions

MC and EM initiated, designed and supervised the study, analysed the results and wrote the manuscript. DM, DC, GR and CP performed the study. GM, GN and SL provided general advice and contributed to data interpretation. All Authors read, discussed, contributed to and approved the final manuscript.

## Pre-publication history

The pre-publication history for this paper can be accessed here:


